# Using cost-effectiveness analysis to support policy change: varenicline and nicotine replacement therapy for smoking cessation in Jordan

**DOI:** 10.1186/s40545-020-00270-y

**Published:** 2020-10-27

**Authors:** Saba Madae’en, Nour Obeidat, Mohammad Adeinat

**Affiliations:** 1 Department of Biopharmaceutical and Clinical Pharmacy, University of Jordan, Amman, 11941 Jordan; 2 King Hussein Cancer Centre, Amman, Jordan; 3 Department of Economics, University of Jordan, Amman, Jordan

**Keywords:** Smoking cessation, Varenicline, Cost-effectiveness, Nicotine replacement therapy, Jordan

## Abstract

**Background:**

Smoking cessation pharmacotherapies (SCPs) have been established as cost-effective for the treatment of tobacco use disorder across a variety of settings. In Jordan, a resource-constrained country where smoking rates rank at one of the highest globally, the cost-effectiveness of SCPs has not yet been quantified. The lack of information about the value of SCPs has contributed to low demand for them (from public and private payers) and consequently low availability of these medications. The aim of this study was to simulate—in a hypothetical cohort of Jordanian smokers—the clinical and economic impact of using two smoking cessation regimens and to generate cost-effectiveness values that can support policy changes to avail smoking cessation medication in a country burdened with heavy tobacco use.

**Methods:**

We employed a similar approach to a widely used economic model, the Benefits of Smoking Cessation on Outcomes (BENESCO) model. A hypothetical cohort of Jordanian male smokers aged 30 to 70 years and making a quit attempt using either a varenicline regimen or a nicotine replacement therapy (NRT) regimen were followed over time (until reaching 70 years of age). Markov simulations were run for the cohort, and life years gained were computed for each arm (compared to no intervention). Drug costs, prevalence of smoking, and population life expectancies were based on Jordanian data. Efficacy data were obtained from the literature. Incremental cost-effectiveness ratios as well as the potential budgetary impact of employing these regimens were generated. Several parameters were modified in sensitivity analyses to capture potential challenges unique to Jordan and that could impact the results.

**Results:**

For a treatment cohort of 527,118 Jordanian male smokers who intended to quit, 103,970 life years were gained using the varenicline regimen, while 64,030 life years were gained using the NRT regimen (compared to the no-intervention arm of life years). The cost per life year gained was JD1204 ($1696 USD) and JD1342 ($1890 USD) for varenicline and NRT, respectively.

## Background

The age-adjusted prevalence of smoking in Jordan ranks among the highest worldwide, having reached 70.2% in males [1]. Overall, the smoking prevalence is approximately 32%, but among middle-aged males, prevalence rates reach as high as 61% [2]. The negative health consequences of smoking are irrefutable: active smoking is associated with numerous diseases and conditions, including cardiovascular and respiratory diseases, negative reproductive effects in both males and females, rheumatoid arthritis, reduced immune function, overall diminished health, and at least 15 types of cancers [3]. Globally, developing countries are particularly hard-hit as a result of scarce resources to manage smoking-induced morbidity and mortality [4, 5]. Availing smoking cessation pharmacotherapies (SCPs) to assist smokers in quitting can considerably alleviates this burden [4].

In Jordan, only 20% of smokers in Jordan reported receiving medical advice to quit smoking, while approximately 63% had tried but failed to quit [6]. Tobacco-dependence treatment guidelines have since been established for the country [7], but bulk procurement of first-line, smoking cessation medications [approved by the US Food and Drug Administration (FDA) as well as the European Medicines Agency] does not take place. Thus, these medications (oral agents varenicline and bupropion, medications that alter the release or uptake of dopamine in the brain and can partially block nicotinic receptors; and nicotine replacement therapies in the forms of gum, lozenge or patch, which provide nicotine in lower concentrations than that found in cigarettes) [8] are not available in a consistent and sufficient quantity in the Jordanian public sector [7]. This is unfortunate, given the evidence that SCPs work. In one of the most recent global clinical trials comparing multiple SCPs, varenicline was associated with 6-month abstinence rates of approximately 25%, while bupropion or NRTs (transdermal patches) were associated with abstinence rates of approximately 18% [9]. Evidence also indicates that extending the use of or combining different SCPs can increase abstinence rates [8]. Practically, the availability of a selection of SCPs, rather than only one medication (for example, only varenicline), is critical to the treatment process. This is because the selection of a SCP is tailored to patient preference as well as patient response [10] and may need to be modified during treatment.

One of the reasons contributing to the lack of urgency to promote SCPs on a national level has been the limited evidence on the pharmacoeconomic value of SCPs from the Jordanian healthcare perspective. While the cost-effectiveness of SCPs has been demonstrated in various Western settings [11], the results cannot be directly extrapolated to developing countries such as Jordan due to differences in prevalence of smoking, population distributions, patient characteristics, and drug pricing.

Our research aims to fill a major information gap by assessing the potential clinical and economic impact of two FDA-approved SCPs from a Jordanian public payer perspective, and accordingly quantifying the cost-effectiveness values for these SCPs if used in Jordan. We were specifically interested in examining whether or not these medications—if employed in a Jordanian population and using Jordanian drug prices—would yield comparable health benefits (quantity of life years gained) as reported in literature from other countries. We also were interested in estimating the budget impact of using such medications in Jordan.

## Methods

### Study design

We conducted a cost-effectiveness analysis on a hypothetical cohort of Jordanian smokers representing the current age distribution of the Jordanian population [12, 13].

### Modeling approach and comparators

Many models have been used to test the cost-effectiveness of SCPs [11]. We referred to the approach used by specific studies [14, 15]. Specifically, we used a Markov model which allowed for one treatment event (at year one) and would follow the cohort until 70 years of age. We thus modeled smoking cessation in a dynamic (time-varying manner) while also taking into account the risks for relapse after 52 weeks.

Three possible intervention arms were used: treatment with varenicline for 3 months, treatment with NRTs (combined patch and gum) for 3 months, and physician advice over three visits with no medications. Both varenicline and the NRT forms selected for our model are approved by the Jordanian FDA (JFDA) for purchasing and use.

**Fig. 1:**
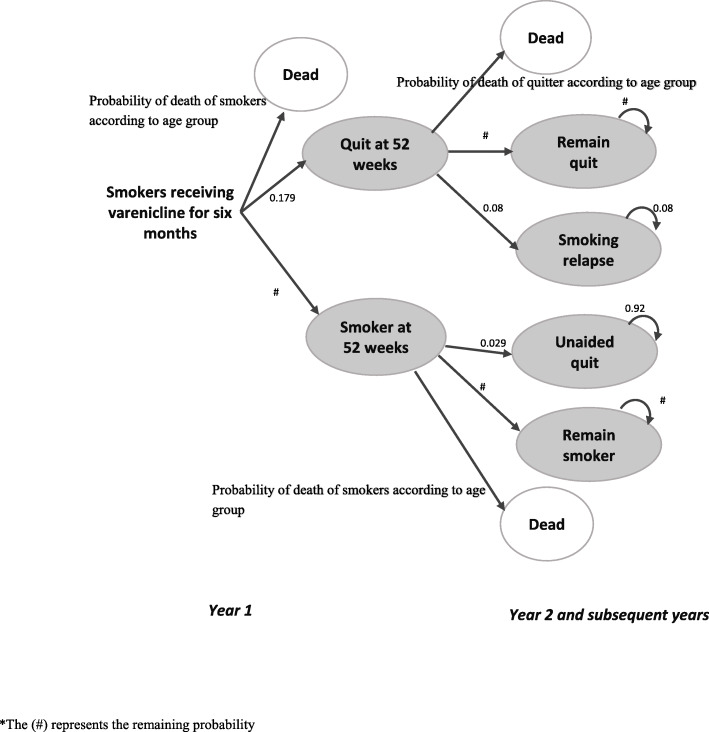
Schematic of model for a single arm (e.g., varenicline users). The number sign represents the remaining probability

Cycles of 1-year length were used. For each arm, the model included three states at the end of the first year: continuing to smoke at 52 weeks, being a quitter at 52 weeks, or being dead. Subsequent to this year, smokers could stop smoking unaided, continue to smoke, or die; quitters could remain abstinent, relapse to smoking, or die. A time horizon of 40 years or up to 70 years of age was used. A schematic of this is presented in Fig. 1, which depicts the cycle for the first year and then subsequent years for varenicline users, and the probabilities of each state transition.

### Perspective

The analysis used the Jordanian Ministry of Health (public payer) perspective.

### Population

This hypothetical study cohort was composed of male smokers in Jordan aged 30 years or older who intended to quit smoking. We chose the male population due to the substantially higher prevalence of smoking among males in the country [2]. Specifically, we constructed the final cohort using 2016 population estimates of Jordan [13]. We applied smoking prevalence rates by male age-groups [2] as well as proportions of smokers willing to quit smoking [16] to the 2016 population estimates to generate the starting population of Jordanian male smokers who would be receiving a smoking cessation intervention.

### Costs

We included only medication costs and costs for physician visits. Similar to other studies [9, 17, 18], we did not include the adverse events of smoking cessation medications due to their relative safety, and due to the fact that incidence of adverse events (such as nausea and headaches) usually do not require major medical interventions.

### Time horizon

We followed each age group up to 70 years of age. Annual cycles were included in the model to reflect the annual probabilities of death.

### Discount rate employed

An annual 3% discount rate was used for all inputs and rewards. The issue of discount rate selected continues to be debated, but we selected the lower of the two most commonly used rates in the literature [19].

### Effectiveness of smoking cessation interventions

We used similar 52-week abstinence rates for the smoking cessation regimens used by Baker and Pietri [20] and which were imputed from the latest randomized controlled EAGLES trial [9]. The effectiveness of brief counseling was an approximate value (5%) taken from key reviews [21, 22].

### Benefits of smoking cessation

We used life years gained as the main benefit of smoking cessation. We did not have sufficient information specific to the Jordanian healthcare setting to generate morbidity or event-specific costs (such as costs associated with altered rates of tobacco-related diseases). We used Jordanian life table numbers to obtain the death rate for each age group of male Jordanians [13]. We then used Taylor et al.’s study to calculate the hazard ratio of death for smokers in each group, as well as the hazard for death in quitters according to the time since they quit [23].

### Outcomes

We generated life years gained as a result of smoking cessation through medication use in each arm (varenicline and NRTs), compared to no medication use. We also generated the total direct costs incurred as a result of medication use. Finally, costs per life years gained were calculated as a measure of incremental cost-effectiveness.

### Threshold

More than one method has been proposed to determine cost-effectiveness thresholds [24–26]. To address the variable estimates of thresholds, we opted to interpret our results using more than one threshold. A relatively generous threshold of one to three times the gross domestic product (GDP) per capita was considered [24], as was a more conservative threshold that was approximately 0.21 to 0.84 times the GDP [25]. Jordan’s per capita GDP in 2019 was estimated at JD3116 ($4395 USD) [27]. Thus, a generous threshold of JD3116 ($4395 USD) was considered, as was a conservative threshold of JD1636 ($2307 USD) (the midpoint for the range suggested by Woods et al. [25] was used to calculate this). Table 1 summarizes the parameters used in the Markov model.

**Table 1:** Overview of parameters used in the base case analysis of cost-effectiveness of varenicline, combined nicotine replacement therapy, or no pharmacotherapy

Parameter	Value	Reference (if applicable)
Prevalence of smokers per age group	30–39, 61.3%; 40–49, 61.4%; 50–59, 62.3%; 60+, 24.8%	Jaghbir et al. [6]
Prevalence of smokers intending to quit in the next 30 days	49%	Abughosh et al. [16]
Effectiveness (derived 52-week abstinence rates)		Baker and Pietri, Fiore et al., Stead et al. [20–22]
Varenicline	17.9%	
NRTs	13.3%	
No medication (single physician visit)	5.0%	
Probability of remaining abstinent having quit by 52 weeks	95%	Hughes et al. [28]
Risk of relapse after one year of abstinence	8%	Hughes et al. [28]
Unaided quit in subsequent years	2.9%	Jaghbir et al. [6]
Jordanian life table numbers were used to obtain the gender-specific death rate for each age group. We then used Taylor et al.’s study to derive the hazard ratio of death for smokers in each group, plus the hazard for death among quitters according to the time since they quit (Appendix 1)	30–34 years, 0.005 × 1.6875; 35–39 years, 0.007 × 1.6875; 40–44 years, 0.011 × 2.34; 45–49 years, 0.018 × 2.34; 50–54 years, 0.03 × 2.82; 55–59 years, 0.051 × 2.82; 60–64 years, 0.081 × 2.80; 65–69 years, 0.129 × 2.80; 70–74 years, 0.205 × 2.52	WHO and Taylor et al. [1, 23]
Costs of treatment (US dollars)		Pharmaceutical unit prices retrieved from the Jordanian Food and Drug Administration (JFDA) [29]
Varenicline, physician visits (3 months)	270.00	
NRTs, physician visits (3 months)	192.00	
No medication (three physician visits)	21.00	
Discount rate	3%	Attema et al. [19]
Cost-effectiveness threshold	JD3116/$4395 (generous); JD1636/$2307 (conservative)*; JD8000 by WHO	Woods et al. and WHO [24, 25]

*The midpoint was selected for a range (approximately 0.21 to 0.84 times the per capita GDP) calculated by Woods et al.

Analyses were performed using the TreeAge Pro software program [30].

### Sensitivity analyses

We addressed the uncertainty in the base case by varying the values of specific input parameters. Specifically, sensitivity analyses were performed in which we varied medication effectiveness rates (relapse rates) and prescription treatment costs. Parameter uncertainty (variability) was reflected either using 95% confidence intervals or the differences in estimates across studies. The tested parameter intervals are shown in Table 3. Each input was considered in isolation in a deterministic one-way sensitivity analysis for each arm.

In a probabilistic sensitivity analysis, we considered all input uncertainties simultaneously, using Monte Carlo simulation (10,000 simulations in different combinations) to yield a single 95% confidence interval of simulations for varenicline cost per life year saved in comparison to brief counseling.

## Results

### Cost-effectiveness

For a treatment cohort of 527,118 Jordanian male smokers who intended to quit, 103,970 life years were gained using the varenicline regimen, while 64,030 life years were gained using the NRT regimen (compared to the no-intervention arm). The cost per life year gained was JD1204 ($1696 USD) and JD1342 ($1890 USD) for varenicline and NRT, respectively. Results of the base case scenario are presented in Table 2. Overall life years gained from varenicline use was 0.197 life years per smoker intending to quit (being highest at age 30, with 0.26 life years gained) and 0.121 life years gained from nicotine replacement therapy.

**Table 2:** Life years gained and medication costs incurred per treatment arm and age group

	Cost (in JDs)	Life years	Number intending to quit	Cycles
**Age 30–34**				
Varenicline	33,043,140	2,829,590	123,476	40
NRT	23,497,344	2,815,827	123,476	40
Brief counseling	2,570,022	2,797,447	123,476	40
**Age 35–39**				
Varenicline	29,199,690	2,013,904	109,453	35
NRT	20,764,224	2,004,786	109,453	35
Brief counseling	2,271,087	1,986,592	109,453	35
**Age 40–44**				
Varenicline	24997680	1330153	95,062	30
NRT	17776128	1321244	95,062	30
Brief counseling	1944264	1306761	95,062	30
**Age 45–49**				
Varenicline	20,887,200	825,156	80,768	25
NRT	14,853,120	820,792	80,768	25
Brief counseling	1,624,560	812,682	80,768	25
**Age 50–54**				
Varenicline	14668560	408252	59,340	20
NRT	10430976	405324	59,340	20
Brief counseling	1140888	402227	59,340	20
**Age 55–59**				
Varenicline	9,338,760	182,218	40,366	15
NRT	6,640,896	181,688	40,366	15
Brief counseling	726,348	180,711	40,366	15
**Age 60–64**				
Varenicline	2,271,510	32,060	10,883	10
NRT	1,615,296	31,897	10,883	10
Brief counseling	176,613	31,363	10,883	10
**Age 65–70**				
Varenicline	1,342,170	13,045	7770	5
NRT	954,432	12,880	7770	5
Brief counseling	104,391	12,625	7770	5
**Total**				
Varenicline	135,748,710	7,625,378	527,118	180
NRT	96,532,416	7,594,438	527,118	180
Brief counseling	10,558,173	7,530,408	527,118	180

### Population and budget impact

In terms of population impact if, hypothetically, all Jordanians intending to quit smoking in the next month sought a practitioner and were managed with a 3-month course of varenicline or NRTs (rather than brief counseling sessions), 94,970 and 64,030 additional life years would be gained using varenicline or NRTs, respectively. These would cost the MoH (Ministry of Health) ($135,748,710 USD) 96,381,584JD and ($96,532,41 6USD) 68,538,015JD (respectively).

### Sensitivity analysis results

The results of the sensitivity analysis are displayed in Table 3.

**Table 3:** Results of deterministic one-way sensitivity analyses for the cost of life year saved by varenicline compared to nicotine replacement therapy

Variable	Base case value	Range varied	Cost per life year gained in JDs (ICER)
Effectiveness of varenicline	0.179	0.15–0.215	1050–7500
Effectiveness of nicotine replacement therapy	0.133	0.1–0.15	400–1100
Effectiveness of brief counseling	0.076	0.029–0.077	500–2500
Cost of varenicline ($)	270	140–400 JD	Dominant
Cost of NRT ($)	192	90–380 JD	Dominated

The input that had the widest range for cost per life year for varenicline was the effectiveness of varenicline. At an effectiveness rate of 15% for example, the cost per life year saved for varenicline was JD7500 ($10,563 USD) in relation to NRT. When costs were varied due to the anticipation that—in reality—bulk purchasing can result in lower unit costs paid by the Jordanian MoH and that generic formulations may become available, the lowest prices of varenicline or high price of NRTs yielded negative, showing that varenicline dominates.

Probabilistic sensitivity analysis yielded, in simulations for varenicline in comparison to brief counseling, a range for cost per life year saved of (397–7500) JDs.

## Discussion

The aim of our analysis was to compare the population benefits (in terms of life years gained) of using smoking cessation medications in a hypothetical cohort of Jordanian smokers. Our analysis yielded 0.26 and 0.12 additional life years gained per smoker using varenicline or nicotine replacement therapy to quit (relative to brief advise from a healthcare practitioner). These values are comparable to the ranges reported in the literature [14, 20, 31]. The incremental costs per life year gained were JD1204 ($1696 USD) and JD1342 ($1890 USD) for varenicline and NRTs, respectively. Using either cost-effectiveness threshold, we concluded that provision of varenicline is a cost-effective intervention, while provision of NRTs is likely to also be cost-effective (given our thresholds were based on a range).

Our cost-effectiveness estimates for both smoking cessation medication regimens are the first to be generated for Jordan, a country in which smoking prevalence now ranks as one of the highest worldwide [1]. Our results are important and confirm that even in a low-resource country such as Jordan, smoking cessation medications are a cost-effective intervention to avail. Furthermore, our results are conservative. We anticipate, if these medications are to become among the list of medications purchased by the Ministry of Health, that these regimens will in reality be far more cost-effective. Our drug prices in the analysis were based on values listed in Jordan’s FDA, which lists wholesale and pharmacy prices for medications when they are first registered [29]. In reality, due to the government’s bulk purchasing of medications through Jordan’s Joint Procurement Department (JPD), unit costs of medications are substantially lower than the listed wholesale and pharmacy prices: for example, in 2016, the JPD reported purchasing medications at approximately 40% of their market price [32]. Furthermore, given the fact that Jordan’s JPD is likely to acquire smoking cessation medications at much lower prices than those initially listed (market price), the budget impact of using smoking cessation medications is also going to be much lower than our estimated impact of JD135,748,710 ($96,381,584 USD) (for varenicline) and JD96,532,416 ($68,538,015 USD) (for NRTs). Finally, with regard to this matter in particular, the potential for generic bupropion, cytisine, and (in the future) generic varenicline to be availed further supports the feasibility of providing smoking cessation medications on a national level.

Treating tobacco dependence is a particularly critical clinical service for Jordan, given the burden of smoking the country faces. Such an intervention is important for primary, secondary, and tertiary disease prevention and control. In fact, treating tobacco dependence is more effective than other preventive services that tend to be more widely promoted [28]. For example, the number needed to treat to avoid one death (NNTs) in the case of a basic smoking cessation service with 6% effectiveness is 67, and the NNT for a more intensive service with 12% effectiveness is 22. These numbers compare very favorably to the NNTs of preventive services such as lifetime treatment with daily aspirin (NNT 40 to prevent an early death from heart disease), statins use (NNT 71), or mammograms to prevent cancer deaths in women aged 50 to 59 (number needed to screen, 351) [28, 33, 34].

Our study limitations largely stem from the lack of clinical and disease-specific economic data applicable to Jordan. We adopted a simplistic approach in our modeling and did not account for changing transition probabilities for events such as changing interest in quitting. We did not account for clinical outcomes preceding death, such as incidence and costs of tobacco-related diseases, and did not include indirect costs of smoking such as lost productivity due to morbidity or mortality. We also assumed that the effectiveness of smoking cessation medications in the literature is generalizable to the Jordanian population. Furthermore, we did not simulate our cohort further than 70 years of age, because we did not have data beyond this age group. Finally, due to the on-going debate about what cost-effectiveness thresholds should be used in evaluating interventions, it is difficult to decisively make statements about the cost-effectiveness of either varenicline or NRTs, although we draw scenarios in our sensitivity analysis that strongly suggest that, in reality (with changing unit costs due to the government’s procurement process), the use of these regimens will be very cost-effective.

Despite these limitations, our study is the first to our knowledge to showcase one aspect of the value of smoking cessation medication (life years gained). Our findings can be used in strengthening the dialog about the value of smoking cessation medications in clinical practice.

## Supplementary information

**Additional file 1: Appendix 1.** Jordanian ex-smokers’ probability of death*

## Data Availability

All data generated or analyzed during this study are included in this published article [and its supplementary information files].

## Abbreviations

SCPs: Smoking cessation pharmacotherapies
NRT: Nicotine replacement therapy
BENESCO: The Benefits of Smoking Cessation on Outcomes
FDA: Food and Drug Administration
GDP: Gross domestic product
JPD: Joint Procurement Department
MoH: Ministry of Health
NNT: Number needed to treat to avoid one death
WHO: World Health Organization
