# Supplementary material for: Using cost-effectiveness analysis to support policy change: varenicline and nicotine replacement therapy for smoking cessation in Jordan
Source: J Pharm Policy Pract. 2020 Oct 27;13:65. doi: 10.1186/s40545-020-00270-y (PMC7590594; doi:10.1186/s40545-020-00270-y)
Supplement: Supplementary file 1 — Additional file 1: Appendix 1. Jordanian ex-smokers’ probability of death* [file 40545_2020_270_MOESM1_ESM.docx]

Appendix 1: Jordanian ex-smokers’ probability of death*

|  | **Time Since Smoker Quit (in years)** | | | |
| --- | --- | --- | --- | --- |
| **Age Group** | **3–5** | **6–10** | **11–15** | **16+** |
| 30–34 | 1.29 * 0.005 (0.00645) |  |  |  |
| 35–39 |  | 1.46 * 0.007 (0.01022) |  |  |
| 40–44 |  |  | 0.93 * 0.011 (0.01023) |  |
| 45–49 |  |  |  | 0.95 * 0.018 (0.01615) |
| 50–54 |  |  |  | 1.13 * 0.03 (0.0339) |
| 55–59 |  |  |  | 1.13 * 0.051 (0.05763) |
| 60–64 |  |  |  | 1.23 * 0.081 (0.09963) |
| 65–69 |  |  |  | 1.23 * 0.129 (0.15867) |
| 70–74 |  |  |  | 1.32 * 0.205 (0.2706) |
| 35–39 | 1.29 * 0.007 (0.00903) |  |  |  |
| 40–44 |  | 1.46 * 0.011 (0.01606) |  |  |
| 45–49 |  |  | 0.93 * 0.018 (0.01674) |  |
| 50–54 |  |  |  | 1.13 * 0.03 (0.0339) |
| 55–59 |  |  |  | 1.13 * 0.051 (0.05763) |
| 60–64 |  |  |  | 1.23 * 0.081 (0.09963) |
| 65–69 |  |  |  | 1.23 * 0.129 (0.15867) |
| 70–74 |  |  |  | 1.32 * 0.205 (0.2706) |
| 40–44 | 1.29 * 0.011 (0.01419) |  |  |  |
| 45–49 |  | 1.46 * 0.018 (0.02628) |  |  |
| 50–54 |  |  | 1.5 * 0.03 (0.045) |  |
| 55–59 |  |  |  | 1.13 * 0.051(0.0339) |
| 60–64 |  |  |  | 1.23 * 0.081(0.099) |
| 65–69 |  |  |  | 1.23 * 0.129(0.15867) |
| 70–74 |  |  |  | 1.32 * 0.205(0.2706) |
| 45–49 | 1.29 * 0.018 (0.02322) |  |  |  |
| 50–54 |  | 1.86 * 0.03 (0.0558) |  |  |
| 55–59 |  |  | 1.5 * 0.051 (0.0765) |  |
| 60–64 |  |  |  | 1.23 * 0.081(0.099) |
| 65–69 |  |  |  | 1.23 * 0.129(0.1586) |
| 70–74 |  |  |  | 1.32 * 0.205 |
| 50–54 | 1.93 * 0.03 (0.0579) |  |  |  |
| 55–59 |  | 1.86 * 0.051 (0.09486) |  |  |
| 60–64 |  |  | 1.75 * 0.081 (0.14175) |  |
| 65–69 |  |  |  | 1.23 * 0.129 |
| 70–74 |  |  |  | 1.32 * 0.205 |
| 55–59 | 1.93 * 0.051 (0.09843) |  |  |  |
| 60–64 |  | 2.17 * 0.081 (0.17577) |  |  |
| 65–69 |  |  | 1.75 * 0.129 (0.22575) |  |
| 70–74 |  |  |  | 1.32 * 0.205 |
| 60–64 | 2.13 * 0.081 (0.17253) |  |  |  |
| 65–69 |  | 2.17 * 0.129 (0.27993) |  |  |
| 70–74 |  |  | 1.92 * 0.205 (0.3936) |  |
| 65–69 | 2.13 * 0.129 (0.27477) |  |  |  |
| 70–74 |  | 2.08 * 0.205 (0.4264) |  |  |
| 70–74 | 1.98 * 0.205 (0.4059) |  |  |  |

**For ex-smoker, life years only become gained after 3 to 5 years in ex-smoking*
